# P04-09 Clustering of reported activity destinations and use of active transport among older adults

**DOI:** 10.1093/eurpub/ckac095.063

**Published:** 2022-08-29

**Authors:** Kirsi E Keskinen, Essi-Mari Tuomola, Taina Rantanen, Erja Portegijs

**Affiliations:** 1 Faculty of Sport and Health Sciences and Gerontology Research Center, University of Jyväskylä, Jyväskylä, Finland; 2 School of Resource Wisdom, University of Jyväskylä, Jyväskylä, Finland; 3 Center for Human Movement Sciences, University Medical Center Groningen, University of Groningen, Groningen, The Netherlands

**Keywords:** mobility, walking, urban, built environment, GIS

## Abstract

**Background:**

Conducting everyday activities out-of-home may accumulate a large share of older adults' daily physical, especially if active transportation is used. Environmental features in home neighborhood may motivate for higher physical activity, but the role of features around destinations is less known. Our goal was to study 1) clustering of older adults' reported activity destinations, and 2) whether transport mode to a destination was associated with characteristics of destination clusters.

**Methods:**

Data comprise AGNES study participants (901 community-dwelling people aged 75-85 years living in city of Jyväskylä, Finland; 57% women) combined with geospatial data. Using digital mapping, participants located frequently used destinations for shopping, services, and social and spiritual activities on a map, and reported transport mode (active/passive) for each. Geographic information system was used to define distance from home to each destination, to identify spatially clustered destination areas, and to assess destination areas' characteristics (urban location, intersection density, nature versatility, and the proportion of reported social/spiritual destinations of all destinations in the area). Based on their characteristics, destination areas were hierarchically categorized to area types. In mixed model, active transportation (vs. passive) was regressed for area type and adjusted for distance, car use possibility, walking difficulty in 2km, age, sex, and MMSE score.

**Results:**

Of reported destinations within 2km from home (1278 destinations for 642 participants), 81% clustered spatially in 23 destination areas and 19% remained separate. Hierarchical clustering resulted three area types: 1) city centre (versatile activities and nature), 2) less serviced areas (versatile activities and less nature), 3) shopping areas (shopping/service activities and less nature). The proportion of destinations visited using active transportation was 63% in city centre, 68% in less serviced areas, 69% in shopping areas, and 56% for separate destinations outside the areas. Based on mixed model results, the odds for active transport use were higher when destinations located in city centre (OR = 4.8, 95%CI 1.3-17.0) or in shopping areas (OR = 11.9, 95%CI 2.6-55.6) compared to visiting locations outside spatially clustered destination areas.

**Conclusion:**

Majority of older adults' activity destinations locate as spatially clustered. Varied destinations close to one another may promote active transport.

